# Tracking Nitrogen Source Using δ^15^N Reveals Human and Agricultural Drivers of Seagrass Degradation across the British Isles

**DOI:** 10.3389/fpls.2018.00133

**Published:** 2018-02-06

**Authors:** Benjamin L. Jones, Leanne C. Cullen-Unsworth, Richard K. F. Unsworth

**Affiliations:** ^1^Sustainable Places Research Institute, Cardiff University, Cardiff, United Kingdom; ^2^Project Seagrass, Cardiff, United Kingdom; ^3^Seagrass Ecosystem Research Group, College of Science, Wallace Building, Swansea University, Swansea, United Kingdom

**Keywords:** stable isotope analysis, sewage signals, nitrogen, seagrass, agriculture

## Abstract

Excess nutrients shift the ecological balance of coastal ecosystems, and this eutrophication is an increasing problem across the globe. Nutrient levels may be routinely measured, but monitoring rarely attempts to determine the source of these nutrients, even though bio-indicators are available. Nitrogen stable isotope analysis in biota is one such bio-indicator, but across the British Isles, this is rarely used. In this study, we provide the first quantitative evidence of the anthropogenic drivers of reduced water quality surrounding seagrass meadows throughout the British Isles using the stable nitrogen isotope δ^15^N. The values of δ^15^N ranged from 3.15 to 20.16‰ (Mean ± SD = 8.69 ± 3.50‰), and were high within the Thames Basin suggesting a significant influx of urban sewage and livestock effluent into the system. Our study provides a rapid ‘snapshot’ indicating that many seagrass meadows in the British Isles are under anthropogenic stress given the widespread inefficiencies of current sewage treatment and farming practices. Ten of the 11 seagrass meadows sampled are within European marine protected sites. The 10 sites all contained seagrass contaminated by nutrients of a human and livestock waste origin leading us to question whether generic blanket protection is working for seagrasses in the United Kingdom. Infrastructure changes will be required if we are to develop strategic wastewater management plans that are effective in the long-term at protecting our designated Special Areas of Conservation. Currently, sewage pollution is a concealed issue; little information exists and is not readily accessible to members of the public.

## Introduction

The influx of nitrogen is critical in shaping the structure and function of aquatic ecosystems across multiple levels (Ryther and Dunstan, 1971; Carpenter et al., 1998; Rabalais, 2002; Conley et al., 2009), but the magnitude of this influx has been immensely altered through the actions of humankind (Vitousek et al., 1997a,b; Galloway and Cowling, 2002; Galloway et al., 2004; Canfield et al., 2010). As a result, available nitrogen in aquatic ecosystems has risen significantly over the last century with far-reaching environmental impacts including changes to productivity, species composition, habitat status, and diversity (Cloern, 2001; Deegan, 2002; Rabalais et al., 2009). While increased nitrogen input is just one element of a suite of anthropogenic stressors that effects near-shore environments, it is generally the result of land-use changes, coupled with increasing coastal populations, and intense animal agriculture (Baden et al., 1990; Mallin and Cahoon, 2003; Lotze et al., 2006; Howarth, 2008; Rabalais et al., 2009; Jones and Unsworth, 2016; Unsworth et al., 2016). Evidence suggests that eutrophication driven by increased nutrient inputs presents the biggest threat to seagrass meadows (Waycott et al., 2009; Unsworth et al., 2015).

The seagrass *Zostera marina* forms a highly productive habitat within the temperate coastal ecosystems of the Northern Hemisphere (den Hartog, 1970). However, in recent decades meadows of *Z. marina* have declined in size and health (Short and Wyllie-Echeverria, 1996; Orth et al., 2006) with anthropogenic activity generally held accountable (Short and Wyllie-Echeverria, 1996; Waycott et al., 2009). Globally, seagrass is lost at a rate of 7% yr^-1^ (Waycott et al., 2009), and expanding coastal populations will see these declines increase. Gaining a more comprehensive picture of how these habitats are threatened is vital if we are to protect these habitats for the ecosystem services they provide (Cullen-Unsworth et al., 2014) and requires understanding the local drivers of decline to create effective management solutions (Jones and Unsworth, 2016).

The nutrient over-enrichment of seagrass meadows results in primary production shifts, away from seagrass, to fast-growing nutrient-limited epiphytic microalgae (Burkholder et al., 2007). Epiphytes grow on the surface of seagrass leaf tissue (Hauxwell et al., 2001; Burkholder et al., 2007) and their excessive growth is a contributing factor to seagrass degradation globally (McRoy and Goering, 1970; Heck et al., 2000; Hauxwell et al., 2003; Burkholder et al., 2007; Cabaco et al., 2008), causing smothering and reducing the plants ability to photosynthesise.

The nitrogen contained in human and agricultural waste forms a significant component of total nutrient influx into marine environments. As a result, monitoring the presence and effects of sewage [Urban Wastewater Treatment Directive (91/271/EEC); Bathing Water Directive (2006/7/EC)] and agricultural waste [The Water Framework Directive (WFD) (2000/60/EC); Groundwater Daughter Directive (2006/118/EC)] are important activities within the United Kingdom. A recent report, however, suggests that four out of five rivers in England and Wales consistently fail to achieve the WFD rating of ‘good ecological status’ (WWF-UK, 2017) – defined as “a biological community which would be expected in conditions of minimal anthropogenic impact.” This situation is so poor that most waterbodies will not even reach the “good” status by 2027, mandated by the United Kingdom’s inclusion in the WFD.

No routine monitoring programs in the United Kingdom consider one of the most widely used techniques to identify the source of N; stable nitrogen isotope analysis. Dissolved inorganic nitrogen (DIN) inputs to watersheds have different δ^15^N values meaning that the source of N can be easily tracked. Inputs from precipitation (-7 to +1‰), biologically fixed N (∼0‰), and inorganic fertilizers (-3 to +3‰) have lighter δ^15^N signals when compared with those from urban sewage and livestock effluent (+4 to +6‰) (Macko and Ostrom, 1994; McClelland et al., 1997; McClelland and Valiela, 1998; Kjønaas and Wright, 2007; Barnes et al., 2008; Bruland and MacKenzie, 2010). As a result, studies to detect N born from urban sewage and livestock effluent have drawn upon numerous taxa, including algae (Dailer et al., 2010; Fernandes et al., 2012), rooted macrophytes (McClelland et al., 1997; McClelland and Valiela, 1998; Cole et al., 2004; Connolly et al., 2013; Fourqurean et al., 2015; Gorman et al., 2017), invertebrates (Bucci et al., 2007; Carmichael et al., 2008, 2012; Fertig et al., 2009, 2010; Risk et al., 2009), and fish (Jennings et al., 1997; Schlacher et al., 2005; Hoffman et al., 2012).

Seagrass meadows within the British Isles are in a perilous state, threatened by eutrophication (Jones and Unsworth, 2016) and physical disturbance (Unsworth et al., 2017). Using a bio-indicator approach that involved collecting data on seagrass density and morphology alongside analysis of leaf biochemistry (C, N, and P content) (Jones and Unsworth, 2016), we previously provided evidence that seagrass meadows of the British Isles are mostly in poor condition in comparison with global averages, with tissue nitrogen levels on average 75% higher than global values. Such poor status places their long-term resilience in doubt (Unsworth et al., 2015). Although these systems may be in a perilous state, we know nothing of the sources of the suspected elevated nutrient levels in United Kingdom seagrass. We hypothesize that nitrogen inputs are arising from sewage or animal agriculture (Cabana and Rasmussen, 1996), driving a reduction in seagrass health across the British Isles. Collecting data on Nitrates and Nitrites from water samples alone is arguably sufficient for assessing water quality (Lee et al., 2004). Therefore, other available metrics, including seagrass leaf tissue biochemistry could and should be included in monitoring assessments.

In the present study, we investigate the status of seagrass around the British Isles and seek to determine whether elevated nutrient levels result from sewage and agricultural sources. We discuss these findings in the context of the management strategies in place, and their suitability to ensuring seagrass remains resilient into the future.

## Materials and Methods

### Study Sites

**FIGURE 1:**
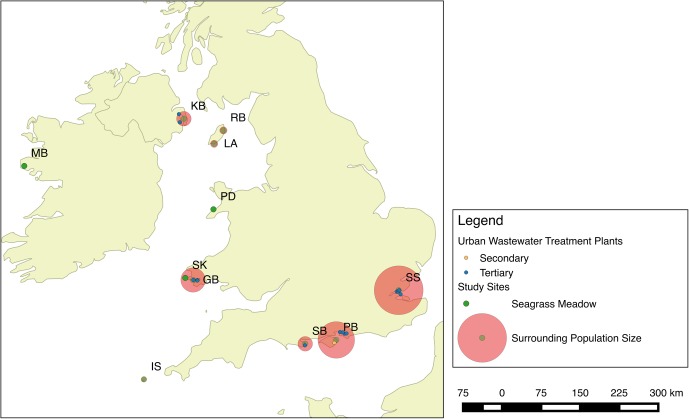
Seagrass meadows were sampled at 11 locations spread throughout the British Isles. These included one site in Ireland, one site in Northern Ireland, three sites in Wales, two sites on the Isle of Man, three sites in England, and seagrass meadows in the Isles of Scilly.

Seagrass meadows at 11 localities around the British Isles were assessed during May–August 2013 (**Figure 1**). At each site, qualitative descriptive information was collected about the perceived presence of anthropogenic impacts to place the data in context; this data is presented in Jones and Unsworth (2016). To apportion these sites into categories of perceived anthropogenic influence, they were *a priori* scored on their status based on five categories (Industry, tourism, agriculture, catchment, and population). Based on values presented in Bruland and MacKenzie (2010), δ^15^N signals are higher where these factors are an impact. A score of five was a site of high potential impacts and zero was a healthy more pristine site displaying no potential impacts (**Table 1**).

**Table 1:** Seagrass sampling locations around the British Isles, their Anthropogenic impact score and perceived health score calculated from data presented in Jones and Unsworth (2016).

Site	Anthropogenic impact score	Seagrass meadow health status
Gelliswick Bay, Wales (GB)	5	Poor
Southend-On-Sea, England (SS)	5	Poor
Priory Bay, Isle of Wight (PB)	4	Moderate
Ramsey Bay, Isle of Man (RB)	4	Moderate
Studland Bay, England (SB)	4	Moderate
Kircubbin Bay, Strangford Lough, Northern Ireland (KB)	4	Poor
Mannin Bay, Ireland (MB)	3	Moderate
Langness, Isle of Man (LA)	2	Moderate
Porthdinllaen, Wales (PD)	2	Poor
Isles of Scilly (IS)	1	Good
Skomer MCZ, Wales (SK)	1	Poor


### Plant Collection, Morphological Measurements, and Seagrass Status

At sampling sites, three haphazardly placed 0.25 m^2^ seagrass plots containing *Z. marina* were sampled. Seagrass sites were within the range of 0–3 m depth. Shoot density counts and percentage cover estimates were taken on site and recorded (McKenzie et al., 2001). All seagrass material within the quadrats was collected for subsequent analysis. Morphological measurements (leaf width and length, number of leaves per shoot) were taken in the laboratory. All epiphytes, where present, were carefully scraped from both sides of the leaf using a microscope slide. Cleaned leaf sections were dried at 60°C for 24 h and ground until homogenous before dry leaf mass was recorded using an Ohaus balance (maximum: 100 g; *d* = 0.1 mg; Switzerland). Similarly, the scraped epiphytes were also dried at 60°C until they were of constant weight before their dry mass was recorded.

**FIGURE 2:**
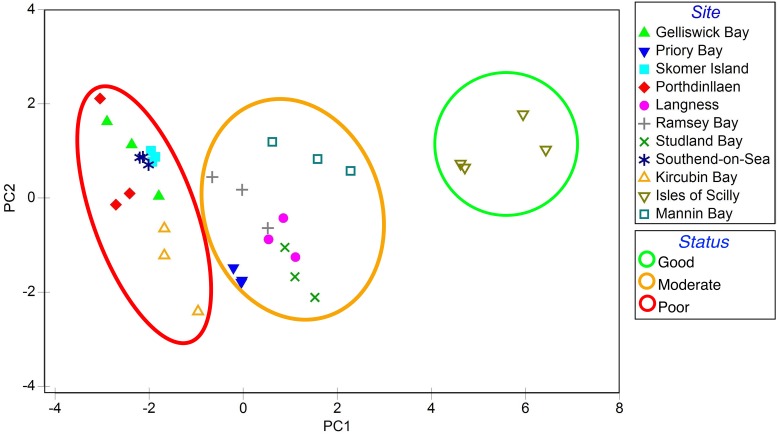
Principal component analysis (PCA) of seagrass characteristics from 11 locations spread throughout the British Isles adapted from Jones and Unsworth (2016). Seagrass meadows were loosely grouped based on PC1 and given a score to reflect status (good, moderate, poor).

Each variable measured different aspects of seagrass status. A Principal Components Analysis (Zar, 1984; Johnson and Gage, 1997; Salita et al., 2003), measuring the spread of sites based on seagrass health and nutrient balance (Jones and Unsworth, 2016), was used to group sites into three health groups (good, moderate, and poor) (**Figure 2**).

### Leaf Content

Seagrass leaf tissue was analyzed for percent nitrogen (N) by weight and atomic percent of ^15^N using a continuous flow isotope ratio mass spectrometer (ANCA SL 20-20, Europa Scientific, Crewe, United Kingdom). Our use of C:N in this study is owing to its use as a robust, early warning indicator of light reduction (McMahon et al., 2013). Isotope values are expressed in the del (δ) notation, δ^15^N, relative to the international standard of atmospheric nitrogen (Connolly et al., 2013):

δ15N(‰)=((N15/N14sample÷N15/N14s⁢tandard)−1)×1000.

### Data Analysis

Statistical analysis was conducted using SPSS. Data were tested for homogeneity of variance and normality. Where data were not normal, log transformations were performed so that data met the assumptions of parametric tests. One-way ANOVA was used to test for differences in δ^15^N values, and proportional δ^15^N values across sites and Pearson’s correlation was used look at the association between δ^15^N and seagrass morphometrics. All values, where described, are reported as mean ± SD.

## Results

### δ^15^N Values across Sites

**FIGURE 3:**
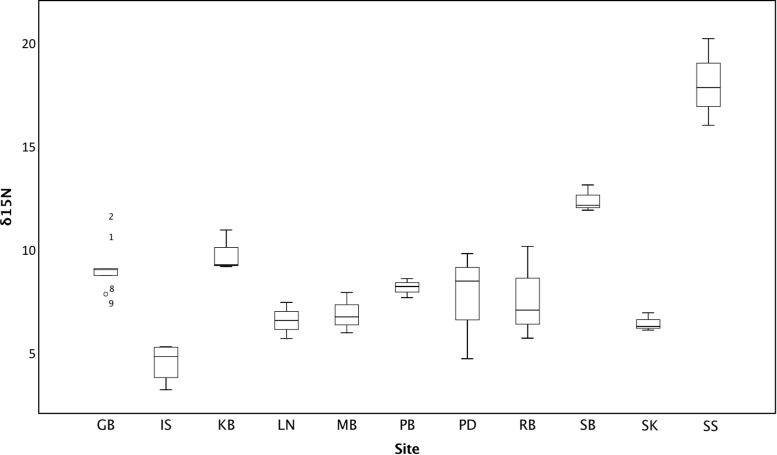
Average leaf δ^15^N (‰) within seagrass at 11 locations spread throughout the British Isles.

**FIGURE 4:**
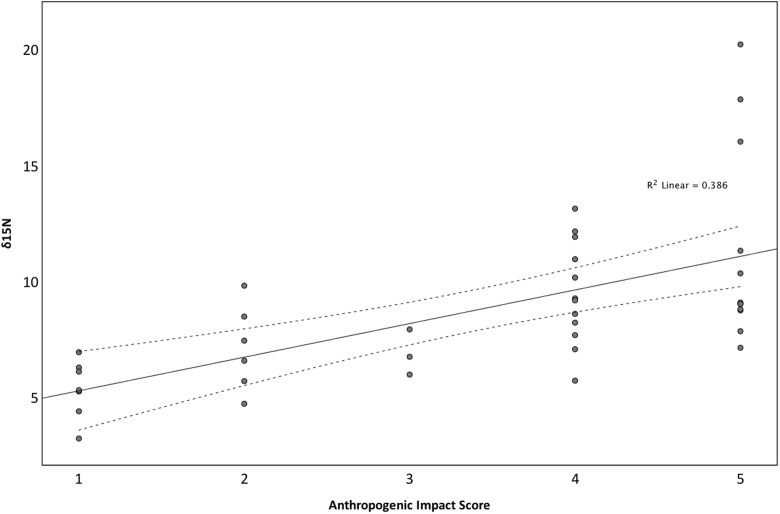
The influence of Anthropogenic Influence Score on δ^15^N values across 11 locations spread throughout the British Isles. Anthropogenic Influence Score was derived from information presented in Jones and Unsworth (2016).

Isotope signals from seagrass leaf tissue differed significantly across 11 sites within the British Isles (*F*_10,29_ = 22.165, *p* < 0.001; **Figure 3**). δ^15^N values ranged from 3.15 to 20.16‰ (8.69 ± 3.50‰), where highest values were recorded from seagrass tissue collected at Southend-on-Sea (17.97 ± 2.1‰), within the Thames waterway and lowest from seagrass tissue collected from the Isles of Scilly (4.47 ± 0.97‰). δ^15^N values were also high in Studland Bay (12.33 ± 0.65‰), Dorset, and Kircubbin Bay (9.73 ± 1.00‰), within the Strangford Lough. Based on data presented in Jones and Unsworth (2016), δ^15^N values were significantly associated with perceived anthropogenic influence (*F*_4,35_ = 7.673, *p* < 0.001; **Figure 4**). Sites with high anthropogenic influence scores (4 and 5; **Table 1**) were characteristic of high δ^15^N values compared with sites of low anthropogenic influence scores (1 and 2; *p* < 0.001).

### Implications for Seagrass Status

**FIGURE 5:**
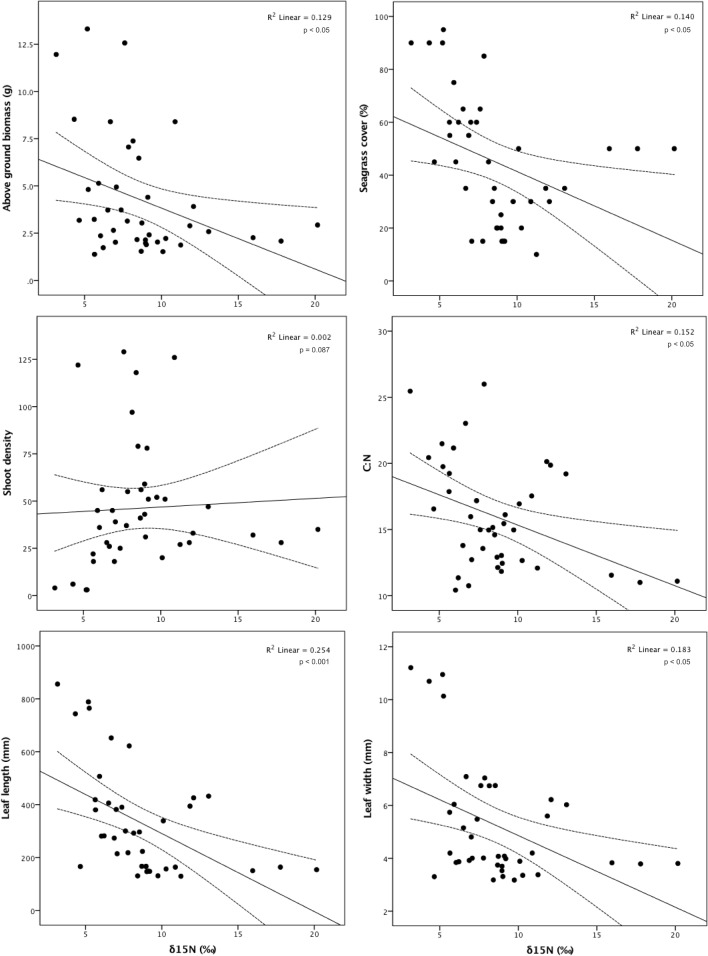
Association between δ^15^N (‰) and aboveground biomass (g), seagrass cover (%), shoot density, C:N ratio, leaf length (mm) and leaf width (mm) for the seagrass, *Zostera marina*, across 11 locations spread throughout the British Isles.

Seagrass meadow status was significantly influenced by δ^15^N (*F*_4,35_ = 7.673, *p* < 0.001). δ^15^N within leaf tissue in the Isles of Scilly, the only site with a ‘good’ status, was significantly lower than δ^15^N values at sites with a moderate (*p* < 0.05) and poor status (*p* < 0.001). However, there was no difference in δ^15^N values between moderate and poor sites (*p* = 0.542). Higher δ^15^N values resulted in significantly lower above ground biomass (*r* = -0.359, *n* = 40, *p* < 0.05), seagrass cover (*r* = -0.375, *n* = 40, *p* < 0.05), shoot biomass (*r* = -0.369, *n* = 40, *p* < 0.05), leaf length (*r* = -0.504, *n* = 40, *p* < 0.001), and leaf width (*r* = -0.428, *n* = 40, *p* < 0.05; **Figure 5**).

However, there was no association between δ^15^N and shoot density (*r* = 0.049, *n* = 40, *p* = 0.765), leaves per shoot (*r* = -0.298, *n* = 34, *p* = 0.087), total epiphyte biomass (*r* = -0.041, *n* = 40, *p* = 0.804), or epiphyte biomass per shoot (*r* = -0.027, *n* = 40, *p* = 0.870). While there was no association between δ^15^N and epiphyte presence, there was however a significant negative association between δ^15^N and the C:N ratio (*r* = -0.390, *n* = 40, *p* < 0.05), suggesting that higher occurrence of δ^15^N leads to light limitation.

## Discussion

Seagrasses form ecologically and economically important and productive coastal habitats, but they are threatened globally (Orth et al., 2006; Waycott et al., 2009; Cullen-Unsworth and Unsworth, 2013). Poor water quality is signaled as one of the leading causes of seagrass loss globally (Ralph et al., 2006; Burkholder et al., 2007; Waycott et al., 2009) but the origins of poor water quality are often poorly investigated. In this study, we provide the first quantitative evidence of the anthropogenic drivers of reduced water quality for seagrass meadows throughout the British Isles. While the data provides a rapid and limited snapshot, it is sufficient to indicate that many seagrass meadows in the British Isles are under anthropogenic stress given the widespread inefficiencies of current sewage and wastewater treatment (WWF-UK, 2017). Nitrogen turnover within seagrass tissue is slow, ranging from weeks to months (Moore and Wetzel, 2000; Lepoint et al., 2002; Connolly et al., 2013) and because these turnover rates determine the time-integrated interval over which seagrasses absorb the nitrogen they have been exposed to Fourqurean et al. (1997) and Fourqurean et al. (2015), the values documented in this study suggest that the wastewater imprint in the receiving seagrass meadows around the British Isles is a persistent feature (WWF-UK, 2017).

Identifying and understanding the prevalence of threats to seagrass at local scales, both direct and indirect, is a fundamental requirement for effective management and to harmonize conservation goals with sustainable economic development. Back-tracing these nutrients from seagrass to source can provide a means for targeted management solutions based on specific local threats. However, the conservation of a specific seagrass meadow is rarely based on the explicit consideration of local threats and drivers, and instead focuses on conserving seagrass as part as a broader management plan incorporating other specific habitats or species. While this may be effective, this way of thinking limits the effectiveness of protection and is conducive to user conflict. For example, 10 of the 11 seagrass meadows examined in this study occurred within European marine protected sites, leading us to question whether generic blanket protection is working for seagrasses in the United Kingdom (Jackson et al., 2016).

While seagrasses are protected under the EU Habitats Directive (92/43/EEC), this directive does not afford protection to all seagrass habitats; it does, however, afford protection through the designation of Special Areas of Conservation (SACs). Within SACs, all activities must be assessed to ascertain whether they would compromise the features of the site. Seagrass should also gain indirect protection from a number of other EU Directives because of its need for good water quality, namely the Urban Wastewater Treatment Directive (91/271/EEC) and the WFD (2000/60/EC). However, a case by case analysis of seagrass leaf tissue here suggests that current protection is inadequate with nutrients from urban sewage and livestock effluent driving eutrophication in numerous *Z. marina* meadows.

Watershed studies reveal that high δ^15^N values (+10 to +25‰) are significantly correlated to a high influx of urban sewage or livestock effluent (Heaton, 1986; Kendall and McDonnell, 2012). As these nutrients are transferred downstream, generally 50% are taken up by aquatic primary consumers and functional feeding groups of primary consumers and are therefore expected to be greatly reduced by the time they reach the marine environment (Galloway et al., 2004; Craig et al., 2008). Given this, high δ^15^N values recorded in seagrass leaf tissue at Southend-on-Sea, Studland Bay, and Kircubbin Bay are of specific serious concern. Based on the Urban Wastewater Treatment Directive, no sites within this study were considered sensitive bathing waters or sensitive eutrophic waters, yet all sites had δ^15^N values of greater than 6‰ except for the Isles of Scilly, suggesting urban sewage and livestock effluent inputs are a prominent feature (Anderson and Fourqurean, 2003; Lepoint et al., 2004). Similarly, other sites in agricultural areas little affected by urban development, such as Porthdinllaen, Mannin Bay, Langness, and Ramsey Bay on the Isle of Man also had significantly higher δ^15^N values. Where livestock waste and manure finds its way into rivers, δ^15^N values are positively correlated with the percentage of land area devoted to animal agriculture (Harrington et al., 1998; Hebert and Wassenaar, 2001; Udy and Bunn, 2001).

### Seagrass Ecosystem Service Provision

This study highlights wider concerns for the ecosystem services that seagrass meadows provide given that over-enrichment results in seagrass degradation (Burkholder et al., 2007) through epiphyte smothering, decreasing light absorption ability (Neverauskas, 1987; Lavery and Vanderklift, 2002; Jones and Unsworth, 2016), and therefore results in the loss of the habitat. Seagrass meadows around the British Isles are known to support populations of juvenile fish of commercial importance (Jackson et al., 2001; Bertelli and Unsworth, 2014; Lilley and Unsworth, 2014). Additionally, evidence suggests that there is a measurable effect of reduced seagrass cover on the abundance and distribution of fauna. Both species composition and species richness are higher in healthier seagrass (High cover, above ground biomass, and larger leaves) indicating increasing habitat value as seagrass complexity increases (McCloskey and Unsworth, 2015).

While N plays a part in regulating seagrass morphometrics, our evidence shows that where δ^15^N values were higher, key seagrass morphometrics were reduced. There is no evidence to suggest that δ^15^N is detrimental to seagrass health thus signaling that N over-enrichment derived from urban sewage and livestock effluent is driving seagrass decline within the United Kingdom. Sites with high inputs of N born from human sewage and livestock effluent were characteristically in a poor state of health with reduced aboveground biomass, lower percentage cover and smaller and thinner leaves. We hypothesize that other elements of urban sewage and livestock effluent may also be entering the system, including heavy metals and bacteria, but this needs further study. Given it exists in a fairly large catchment, seagrass at Gelliswick Bay in the Milford Haven Waterway was somewhat an exception. Despite having high %N values and in a characteristically poor state (Jones and Unsworth, 2016), levels of δ^15^N were relatively low in comparison to other sites existing near to or adjacent to large catchments. It is therefore, probable that much of the N was of inorganic origin. Sub-tidal seagrass at Gelliswick is showing long-term trends of decline (Nagle, 2013), and the evidence here indicates that the probable driver of this is catchment-wide fertilizer use.

While seagrasses are efficient at decreasing pollution, reducing bacteria in water column by up to 50%, this can be at a detriment for protecting themselves from pathogens (Lamb et al., 2017). Ultimately, under stress, the plants are more susceptible to instances of disease. Seagrass collected from Southend-on-Sea had the highest δ^15^N values, and a recent study using plants from the same population identified that nitrate enrichment increased the susceptibility of *Z. marina* to wasting disease (Hughes et al., 2017). What is more trivial given that livestock production is one of the largest drivers of climate change and largest contributor of greenhouse gas emissions worldwide (about 30%) (Tilman et al., 2017), is that meadows of *Z. marina* in these areas, and a key carbon sink (Greiner et al., 2013; Röhr et al., 2016), were in a perilous state with their long-term resilience at risk (Jones and Unsworth, 2016).

Significant progress in the development of a Marine Protected Area network has occurred in some parts of the British Isles in the last decade. The finding of the present study highlights that management needs to consider catchment issues in addition to the creation of MPAs. Evidence from other parts of the world (Quiros et al., 2017) illustrates that creating marine protected areas alone is insufficient to protect seagrass, as the major threats arise from poor water quality.

Unquestionably, the nutrient enrichment of coastal marine waters of the British Isles is of serious concern, with potential system-wide consequences. Seagrass meadows may already be nearing a point of no-return in some cases, with leaf N 75% higher than global values. Solving this issue extends beyond “protecting” seagrasses within a SAC legislation and challenges the way we think about marine protection. Serious infrastructure changes are key to this if we are to develop strategic wastewater management plans that are effective in the long-term.

## Author Contributions

BJ and RU conceived the study; BJ conducted the study and analyzed the results; BJ, RU, and LC-U wrote and reviewed the manuscript.

## Conflict of Interest Statement

The authors declare that the research was conducted in the absence of any commercial or financial relationships that could be construed as a potential conflict of interest.
